# Quantifying thoracolumbar fascia deformation to discriminate acute low back pain patients and healthy individuals using ultrasound

**DOI:** 10.1038/s41598-024-70982-7

**Published:** 2024-08-29

**Authors:** Andreas Brandl, Jan Wilke, Thomas Horstmann, Rüdiger Reer, Christoph Egner, Tobias Schmidt, Robert Schleip

**Affiliations:** 1 Department of Sports Medicine, Institute for Human Movement Science, Faculty for Psychology and Human Movement Science, University of Hamburg, Hamburg, Germany; 2 Conservative and Rehabilitative Orthopedics, TUM School of Medicine and Health, Technical University of Munich, Munich, Germany; 3 Vienna School of Osteopathy, Vienna, Austria; 4 Department of Movement Sciences, University of Klagenfurt, Klagenfurt, Austria; 5 Department for Medical Professions, Diploma Hochschule, Bad Sooden-Allendorf, Germany; 6 Institute of Interdisciplinary Exercise Science and Sports Medicine, MSH Medical School Hamburg, Hamburg, Germany

**Keywords:** Thoracolumbar fascia deformation, Ultrasound, Reliability, Acute low back pain, Health care, Risk factors

## Abstract

Reduced shear strain and deformability of the thoracolumbar fascia has been linked to low back pain. A number of ultrasound examination methods have been developed for laboratory rather than clinical practice. The aim of this study was to examine the reliability and discriminative validity (patients vs. healthy individuals) of an ultrasound (US) measurement method for the quantification of thoracolumbar fascia deformation (TLFD). A cross-sectional study with US assessment and rater blinding was conducted in a manual therapy clinic and a university laboratory. 16 acute low back pain (aLBP) patients and 15 healthy individuals performed a standardized trunk extension task. US measurements of TLFD were carried out independently by two raters by imaging the TLF in the starting and ending positions of the movement. Intra-rater and inter-rater reliability were calculated using intraclass correlation coefficients (ICCs) and minimal detectable changes (MDC) were calculated. Receiver operating characteristic (ROC) curve analysis was used to determine the cut-off for TLFD to discriminate the study groups. Kappa statistics were performed to assess rater agreement in discrimination. Intra-rater reliability was excellent (ICC: .92, MDC: 5.54 mm, *p* < .001) and inter-rater reliability was good (ICC: .78, MDC: 8.70 mm, *p* < .001). The cut-off for TLFD was 6 mm with a sensitivity of 100% and a specificity of 93.75% and the raters agreed moderately (κ = 0.74, *p* < .001) when distinguishing patients and controls. The reliability of the US method for assessing TLFD is moderate to excellent, and the ability to discriminate aLBP patients from healthy individuals is moderate. The method could be used to capture an additional parameter in morphological aLBP screenings.

## Introduction

Low back pain (LBP) is a compelling global problem that is recognized as the leading cause of disability years lived, placing a significant economic demand on healthcare systems worldwide^1^. For example, the 2016 expenditures for LBP and neck pain were USD 134.5 billion in the United States which corresponds to the highest healthcare costs of the country^2^. About one quarter (26%) of the Austrian population stated having suffered from back pain in 2019^3^. In Germany, LBP is the number one disease, accounting for 14% of the years lived with disability^4^. Research on the course of LBP, furthermore, shows worrisome results: only one-third of patients recover after three months, while the majority experience new episodes of LPB within the next year^5^.

In an effort to understand and predict the occurrence of low back pain, clinicians and scientists have aimed to identify relevant risk factors^6–9^. However, most often, no clear cause can be detected and as no linear causal relationship can be established between specific findings like disc disease or radiculopathy and LBP, the multifactorial etiology of LBP has been recognized and widely accepted^10–12^. Notwithstanding, despite the complex pathogenesis of LBP, some predictors have been revealed. Parreira et al.^6^ conducted an umbrella review including 15 systematic reviews of moderate to high quality and identified e.g. a history of LBP, high age, obesity and physical exertion. In particular, the phases of acute LBP (aLBP) in a person's history proved to be a significant risk factor for the progression of LBP, especially in conjunction with impairments in psychosocial health^6,13^.

With regard to morphological aspects, some authors have suggested the possible involvement of micro-injuries and/or swelling in the paraspinal connective tissue and the thoracolumbar fascia (TLF) as a plausible contributor to LBP^14–17^. The TLF is a diamond-shaped, multilayered aponeurotic structure that separates the paraspinal muscles from the muscles of the posterior abdominal wall. Its posterior layer, to which we refer here, is dominated by the insertion of the latissimus dorsi muscle and covers the erector spinae muscles^18^. In a comprehensive cross-sectional study of 930 individuals with aLBP compared to 6953 pain-free individuals, a significant association was found between differences in paraspinal intramuscular fat and cross-sectional area^19^, which was reported to be associated with an increase in TLF thickness^20^.

A variety of studies have emphasized the nociceptive potential of the TLF which is due to a significant proportion of nociceptors, in congruence with an innervation by both A- and C-fibers^16,21^. In addition, cross-sectional studies revealed reduced shearing mobility between the TLF and the epimysium of the erector spinae muscle in LBP patients^14,17,22^. These alterations could be associated with a potential decrease in elasticity, stiffening (increased Young’s modulus), sliding mobility of the TLF^14^ which, in turn, may cause micro-injuries, inflammation^23^, impairment of proprioceptors^24^, and sensitization of nociceptors^25^. These alterations are being investigated to understand the relationship between fascial mechanics and the factors that influence them^14,17,22^. In addition, understanding the interplay between back muscle function and TLF deformation during lumbar spine, pelvis and hip movements is important for the assessment and diagnosis of disorders associated with low back pain that are relevant to clinical practice^26,27^.

In view of the emerging findings of the sensory role of the TLF, a number of ultrasound (US) examination methods have been proposed over the last decade, trying to quantify the TLF’s gliding or deformation (TLFD) characteristics^9,14,28–31^. Most of them require a strict experimental laboratory setting and/or high-priced cutting-edge technology. For example, some studies have been conducted using an electric treatment bench that passively moves the participant during the TLF glide examination. However, a significant amount of manual therapy practices only have treatment benches that are electrically height-adjustable. Some studies also used 3D camera systems for position control systems of the US transducer^14,32^ and complex customized software for image processing^30,33^.

As US measurements of fascial layer sliding are also being introduced as a new option for daily diagnostics in practice^26^, the development of easy-to-apply methods is of interest. The aim of this study was to examine the intra- (in relation to measurement repetition by an experienced tester, as would occur in daily practice) and inter-rater reliability of a clinical non-laboratory method to determine TLFD and test for its ability to discriminate between aLBP patients and healthy individuals. Further, based on that data, standard error of measurement (SEM) and minimal detectable changes (MDC) ought to be calculated.

## Methods

This work was a repeated-measures reliability and discriminative validity study, conducted in accordance with the Guidelines for Reporting Reliability and Agreement Studies (GRRAS)^34^. The study was part of a larger project assessing neuromotor associations of the TLF that was prospectively registered with the German Clinical Trials Register (DRKS00027074). The study has been reviewed and approved by the ethical committee of the Diploma Hochschule, Germany (Nr.1014/2021), has been carried out in accordance with the declaration of Helsinki and has obtained informed consent from the participants^35^.

### Setting and participants

The study was conducted in a manual therapy clinic (southern Germany) and at a university of health professions (eastern Austria) from November to December 2023. As this study is, to the best of the authors' knowledge, the first comprehensive reliability study that includes inter-rater comparisons, we calculated the sample size based on the current state of research. Therefore, the lowest intra-rater reliability (ICC = 0.88) from a cross-sectional study by Pirri et al.^36^, in which TLF thickness was assessed sonographically, and in which 46 healthy individuals and 46 LBP patients participated, was used as the expected reliability. The required sample size (n = 30), assuming a minimum reliability of 0.7, the previously analyzed expected reliability of > 0.88, α = 0.05, 1−ß = 0.8, and two raters was calculated with the “Sample size calculator (web)”^37^, based on the work of Walter et al.^38^.

In addition, we performed a receiver operating characteristic (ROC) analysis of one investigator's data from a previous study using the same US method in 16 healthy participants and 16 LBP patients^26^. We calculated the sample size with the “Sample size – confidence interval for AUROC” calculator^39^ based on the analyzed area under the curve (AUC) of 0.955, a proportion of the sample with disease of 50% and a minimum acceptable AUC of 0.7 (acceptable according to Mandrekar^40^). As this analysis resulted in a sample size of 28, we planned to enroll a total of 30 participants, with approximately 50% being aLBP patients.

The acquisition for aLBP participants was carried out via direct contact, a notice board, and the distribution of information material at the university. The control group was composed of students and associates of a college of health professions. Rater 1 had over 10 years of experience in US assessment of lumbar myofascial tissue. Rater 2 had a total of 5 years of experience in orthopedic US examination, but was not explicitly trained for the study. Both raters were familiarized with the method for half an hour at the beginning of the study on a test person who did not take part in the study and compared their results, as recommended by Patjin^41^. Inclusion and exclusion criteria were collected together with epidemiologic data (gender, age, weight, height) prior to the study.

### Inclusion criteria for the aLBP participants

Inclusion criteria were: aLBP as defined by the European guidelines for the management of aLBP^10^; a minimum score of 10 on the Oswestry disability questionnaire in the German version (ODQ-D)^42^; a minimum aLBP score of 3 on the visual analogue scale (VAS).

### Exclusion criteria

Exclusion criteria were: rheumatic diseases; intake of medication affecting blood coagulation; intake of muscle relaxants; skin changes (e.g. neurodermatitis, psoriasis, urticaria, decubitus ulcers); surgery or other scars in the lumbar region between Th12 and S1; participant age under 18 or over 60 years. Additional exclusion criteria for the control group were either the presence of current LBP or a recent history of LBP (no LBP episodes in the past 5 years; no history of physician visits due to LBP).

### Ultrasound measurement of the deformation of the thoracolumbar fascia

TLFD US measurement using the junction between the latissimus dorsi muscle (LD) and the TLF as an anatomical landmark was previously described by Brandl et al.^26,29^. A detailed description of the ultrasound assessment protocol can be found at https://dx.doi.org/10.17504/protocols.io.eq2lyjbmwlx9/v1.

**Fig. 1:**
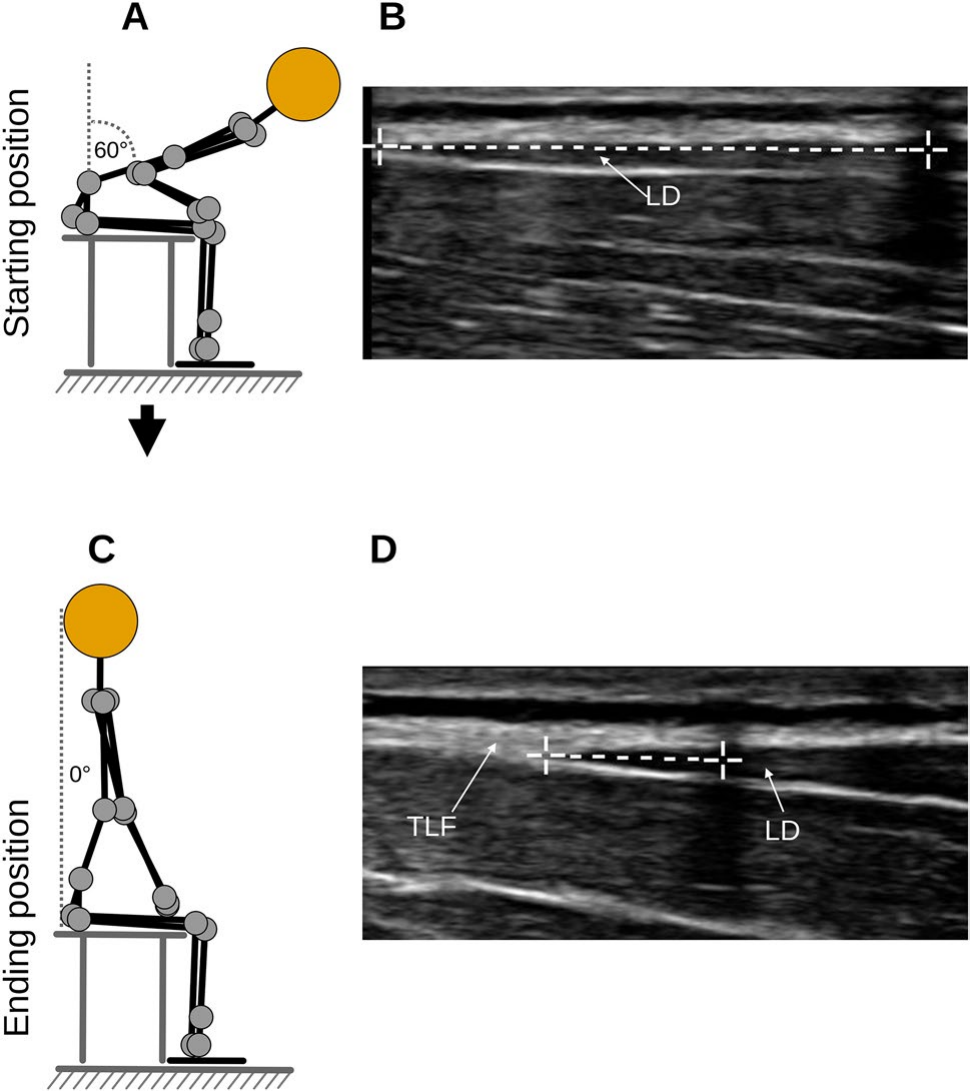
Measurement procedure. (**A**) Flexion phase trunk extension task. (**B**) Measurement time point t_1_. (**C**) Fully extended position of trunk extension task. (**D**) Measurement time point t_2_. The left white cross on the measurement line marks the LD/TLF junction. The white cross on the right marks the center of the artificial reference created by a reflective tape on the skin. TLF, thoracolumbar fascia; LD, latissimus dorsi muscle.

Briefly, the participants sat on a treatment table, bending the trunk until the examiner determined an approximate flexion angle of 60 degrees (starting position), and here, a static US image was taken (Philips Lumify linear transducer L12-4, 12 MHz; Philips Ultrasound Inc., Bothell, WA; Fig. 1A, B). Participants then extended the trunk to 0 degrees, which was the ending position of the trunk extension task (TET), and again, a static, second US image was taken (Fig. 1)C, D). The distance between the junction of the latissimus dorsi muscle and the TLF and an artificial reference made by a reflective tape on the skin shown in the images was measured (Fig. 1B, D). The difference between the distances of the starting position and the ending position represent the extent of the TLFD (Fig. 1B, D).

### Reliability assessment of the ultrasound measurement

The first rater sonographically recorded the transverse process of L1. Subsequently, the transducer was moved laterally until the junction of the latissimus muscle and the TLF was visible. The transducer was rotated laterally and caudally until the fibers of the latissimus muscle were aligned in parallel and the reflective tape was placed on the skin^32^. The rater then performed the first measurement during TET in the starting and ending positions (t_0_). Afterwards, the transducer and the tape were removed without leaving any visible marks on the skin and a second measurement was conducted after 3 min using the same procedure (t_1_). After one hour, rater 2 also performed the measurements using the same procedure (t_2_). The order in which the participants were measured was randomly assigned^43^ for each measurement procedure. Rater 1 was blinded to the results of his first measurements and the raters were blinded to the ratings of the respective other rater.

The data were analyzed in a separate step by a statistician who was blinded to the origin of the data in terms of group membership (aLBP/control) as well as rater (first/second) and only analyzed coded data in this regard.

### Statistical analysis

All descriptive data were reported as means ± standard deviation (SD) and 95% confidence intervals (95% CI). Group differences were tested using Student’s t-test. Intraclass correlation coefficients (ICC) within rater 1 and between rater 1 and rater 2 and their 95% CI were calculated using a 2-way random-effects model (absolute agreement, ICC_(2,*k*)_, multiple raters, *k* = *2*). Resulting ICC values were interpreted according to Koo^44^ as ‘poor’ (< 0.50), ‘moderate’ (0.50 to 0.75), ‘good’ (0.75 to 0.90) and ‘excellent’ (> 0.90). For relative reliability^45^, the corresponding SEM were estimated using the formula (1)^46^:

1 $$SEM=\text{SD}* \sqrt{\left(1-\text{ICC}\right)}$$

The minimal detectable change (MDC) was estimated by reference to the SEM using the formula (2) ^45^:

2 $$MDC=1.96* \sqrt{2}*\text{SEM}= 1.96* \sqrt{2}*\text{SD}* \sqrt{\left(1-\text{ICC}\right)}$$

Bland–Altman plots were created to provide additional visual information about limits of agreement within rater 1 and between rater 1 and rater 2^47^.

The ROC curve and AUC were used to investigate discriminative validity: We determined the cut-off TLFD to distinguish aLBP patients and healthy participants. The variability of the two raters in binary decisions regarding group membership (aLBP patients/healthy participants) was expressed using the kappa coefficient (κ), Gwet’s AC1 coefficient, their 95% CIs, and the proportion of observed agreement^48^. Resulting values were interpreted according to McHugh^49^ as ‘weak’ (< 0.60), ‘moderate’ (0.60 to 0.79), ‘strong’ (0.80 to 0.90) and ‘almost perfect’ (> 0.90) agreement.

All outcomes met the assumptions for parametric testing (*p* > 0.05). Analyses were performed using Jamovi 2.3 (The jamovi project, https://www.jamovi.org).

## Results

In total, n = 31 individuals (19 females and 12 males) took part in the study. Detailed characteristics of the sample are presented in Table 1. The average time required for the whole TLFD measurement procedure was approximately 10 to 12 min. Table 2 shows the descriptive statistics of the measurements of the two raters.

**Table 1:** Sample characteristics.

	Group	N	Mean	95% confidence Interval		SD	*P *Value
				Lower	Upper		
Sex (woman/men)	aLBP	16	10/6				.886^†^
	CTR	15	9/6				
Age (years)	aLBP	16	44.85	39.23	50.47	10.54	.146*
	CTR	15	39.29	33.65	44.92	10.17	
Height (m)	aLBP	16	1.72	1.68	1.77	0.07	.388*
	CTR	15	1.75	1.71	1.79	0.07	
Weight (kg)	aLBP	16	71.38	65.93	76.82	10.21	.461*
	CTR	15	68.47	62.11	74.82	11.46	
BMI	aLBP	16	24.05	22.20	25.90	3.47	.110*
	CTR	15	22.27	20.94	23.60	2.39	
VAS (mm)	aLBP	16	50.63	39.88	61.37	20.15	
ODQ (0–100)	aLBP	16	49.00	41.11	56.89	14.80	
Pain duration (days)	aLBP	16	7.94	6.02	9.86	3.60	

*aLBP* Acute low back pain patients, *CTR* Control group, *BMI* Body mass index, *ODQ* Oswestry Disability Questionnaire. ^†^ Chi squared test for sex group comparison. * Student’s t-test for group comparisons.

**Table 2:** Descriptive statistics of the deformation of the thoracolumbar fascia.

	Group	N	Mean (mm)	95% confidence interval		SD	*P* Value*
				Lower	Upper		
Rater 1 (t_0_)	aLBP	16	− 0.25	− 2.65	2.14	4.50	< .001
	CTR	15	11.72	9.35	14.10	4.29	
Rater 1 (t_1_)	aLBP	16	− 1.05	− 3.09	0.99	3.83	< .001
	CTR	15	10.87	8.94	12.80	3.49	
Rater 2 (t_2_)	aLBP	16	− 1.57	− 3.26	0.11	3.16	< .001
	CTR	15	7.53	5.47	9.59	3.71	

*aLBP* Acute low back pain patients, *CTR* Control group, t_0_, first measurement; t_1_, second measurement after 3 min; t_2_, third measurement by rater 2. * Student’s t-test for group comparisons.

### Reliability assessment of the ultrasound measurement

**Fig. 2:**
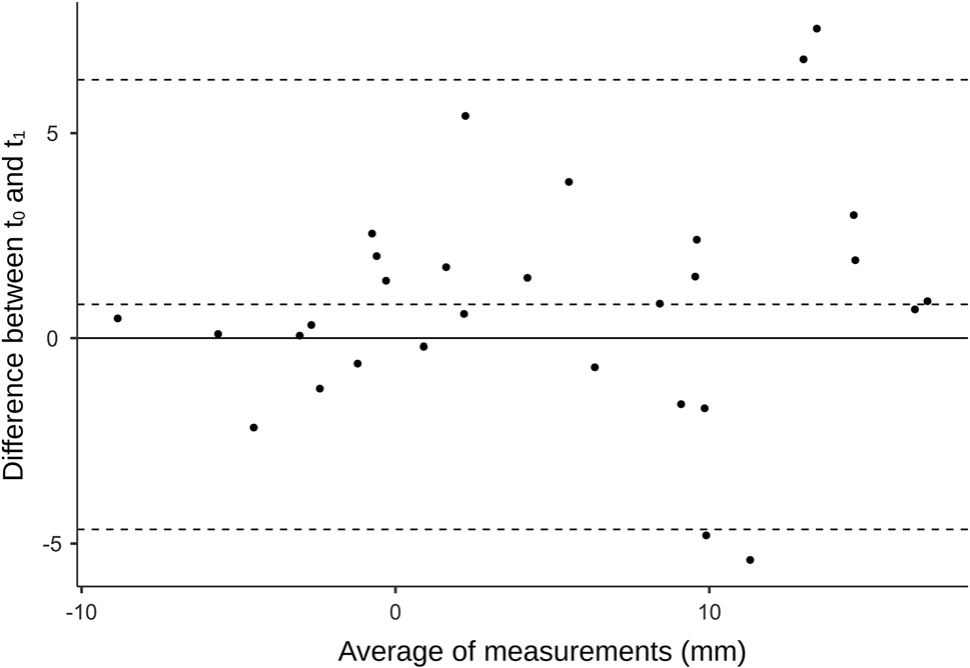
Bland–Altman plot of the intra-rater reliability. t_0_, first measurement of rater 1; t_1_, second measurement of rater 1.

**Fig. 3:**
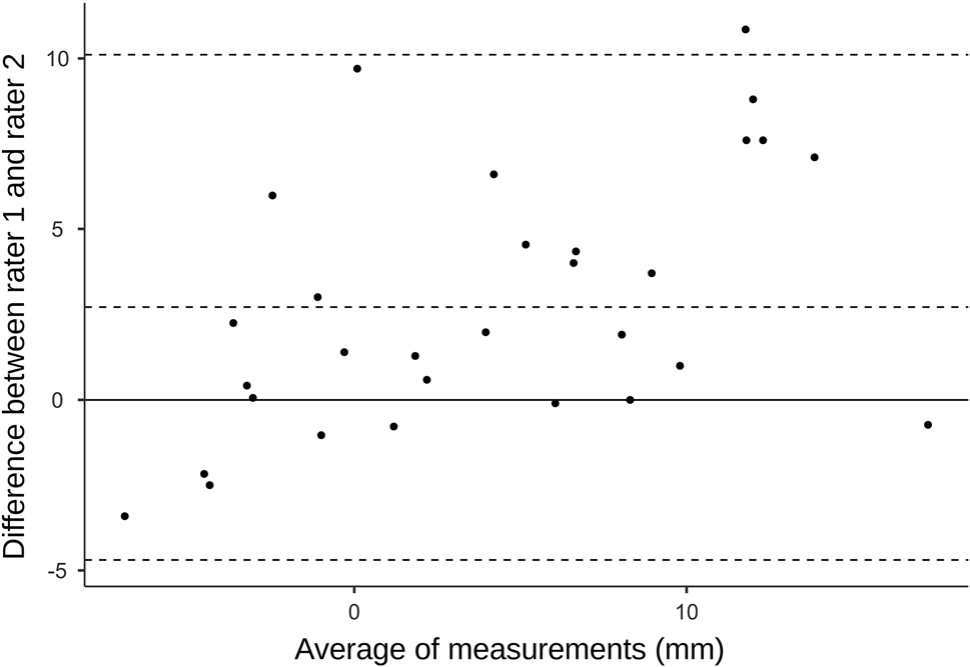
Bland–Altman plot of the inter-rater reliability.

Table 3 shows the detailed reliability results. Intra-rater reliability was excellent (ICC_(2,2)_ = 0.92; *p* < 0.001; SEM = 2 mm; MDC = 5.54 mm) and the inter-rater reliability was good (ICC_(2,2)_ = 0.78; *p* < 0.001; SEM = 3.14 mm; MDC = 8.70 mm). Bland–Altman plots for intra- and inter-rater reliability showed that almost all points were within the limits of agreement (Figs. 2 and 3).

**Table 3:** Reliability.

Typ	ICC	95% CI		F Test				SEM (mm)	MDC (mm)
		Lower	Upper	Value	*df1*	*df2*	Sig		
Intra-rater	.922	.844	.962	26	30.0	29	< .001	2.00	5.54
Inter-rater	.778	.416	.906	11.4	30.0	8.63	< .001	3.14	8.70

95% CI, 95% confidence interval.

### Discrimination of acute lumbar back pain and healthy conditions

**Fig. 4:**
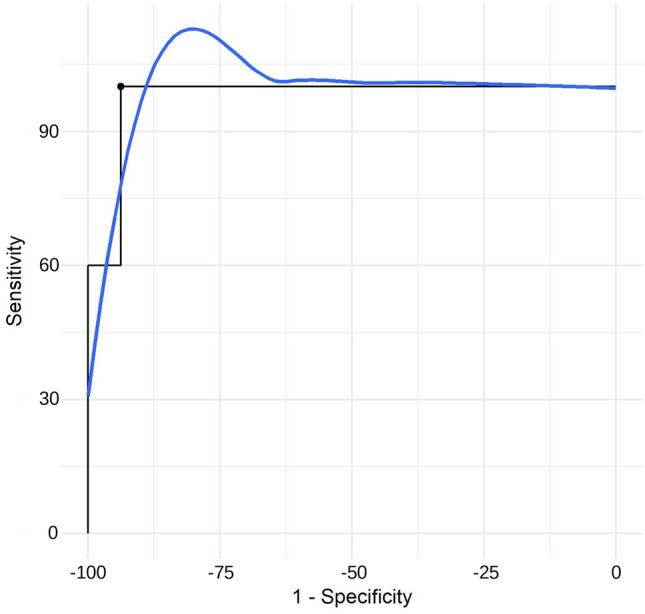
Receiver operating characteristic (ROC) curve. The blue line shows the LOESS smoothing.

The applicability of the TLFD measurement as aLBP discrimination factor showed an AUC value of 0.98, and a corresponding optimal cut-off value of 6 mm (sensitivity 100%, specificity 93.75%), indicating that the model had good discrimination ability (Table 4; Fig. 4).

**Table 4:** Receiver operating characteristic.

Cutpoint	Sensitivity (%)	Specificity (%)	PPV (%)	NPV (%)	Youden’s index	AUC	Metric score
6	100	93.75	93.75	100	0.938	0.975	1.94

*PPV* Positive prediction value, *NPV* Negative prediction value.

### Inter-rater reliability of clinical decision making

The inter-rater agreement between the 2 independent US raters for discrimination of aLBP patients and healthy participants was moderate with respect to Cohen’s Kappa (κ = 0.74, 95% CI = 0.51 to 0.97, z = 4.26, agreement = 87%, *p* =  < 0.001) and Gwet’s AC1 (AC1 = 0.75, 95% CI = 0.50 to 0.99, *p* =  < 0.001). The proportion of observed agreement revealed a sensitivity of 96.9% and a specificity of 83.9%. See Table 5 for descriptive statistics.

**Table 5:** Observed agreement discriminating aLBP/healthy participants.

	*n*
Total	63
Diseased	32
Healthy	31
Positive tests	36
Negative tests	27
True test	57
Wrong test	6

## Discussion

To the authors' knowledge, this is the first article reporting the reliability of an US method to determine TLFD. For this reason, a clinical non-laboratory method was chosen and tested for its ability to discriminate aLBP patients and healthy individuals.

Intra-rater reliability was excellent and inter-rater reliability was good with unbiased repeated measures. The Bland–Altman analysis showed that the mean difference was well below the MDC, indicating that there were no systematic errors and hardly any outliers in the measurements that exceeded the limits of agreement. As expected, the differences increased slightly but remained within the limits of agreement when the TLFDs were at the end of the measurement range. These reliability results are comparable to other US assessments in LBP patients, e.g. of the multifidus muscle thickness or of lumbar motor control tests, which are recommended for diagnosis, demonstrating their suitability as a direct examination technique for the clinical appraisal of TLFD^36,50,51^. While most US studies that quantified the gliding or deformation of the TLF, to the best of our knowledge, many did not test the method for reliability^9,26,29,52,53^. Langevin et al.^14^ determined the intra-rater reliability of their method and found an ICC of 0.98, which was slightly higher than our values. However, they investigated TLF shear strain, and the cross-validity with our new method needs to be clarified in further studies.

Given the current lack of appropriate assessment tools for monitoring treatment effects and the socioeconomic importance of LBP, TLFD may represent an interesting new way to obtain diagnostic information^54^. The MDC of one examiner of 5.54 mm could support the suitability of the US method to a certain extent. However, the 48% higher MDC, determined with two examiners, of 8.7 mm could represent a limit for high-precision measurements in experimental environments^54^.

Analysis of the ROC curve yielded a cut-off point of 6 mm to discriminate aLBP patients from healthy individuals, meaning that in the case of aLBP, the individual has a TLFD of less than 6 mm. This value identified all aLBP patients in the study population (sensitivity of 100%) and also separated 93.75% (specificity) of healthy individuals from them. Agreement between the two independent US raters was moderate. Although 6 of 63 assessments were incorrect, this detection rate is considerably more favorable compared to other paraspinal US assessments in LBP^55–57^ and superior to most manual palpation-based approaches^55^. In the past, US has been reported to be incapable of detecting abnormal echogenicity of paraspinal tissue in LBP^56,58^. Nazarian et al.^56^ therefore reported in a systematic review that the majority of ROC curve analyses were below chance value, with an weak inter-rater agreement of κ = − 0.06, indicating that the overall discriminatory ability of US is worse than the likelihood.

It is likely that these results have hindered the further development of US technologies and methods in the field of LBP diagnosis over the next few years. However, recent research emphasizes the potential of US diagnosis, especially in dynamic examinations of myofascial structures. Cuesta-Vargas et al.^59^ studied LBP patients and healthy controls measured during a TET and classified them based on muscle activation measured with electromyography, pennation angle, and erector spinae muscle thickness. Using this method, they were able to identify 48.5% of LBP patients (sensitivity) and 84.8% of healthy individuals (specificity). The US method for TLFD clearly exceeded these results. Furthermore, the two-dimensional measurement of TLFD may be superior to methods that consider only one dimension such as compressive stiffness or TLF thickness measurements. For the first method, a comprehensive validity and reliability study showed that none of the analyzed instruments were able to detect stiffness changes within the TLF^60^. With regard to the US thickness measurement, it should be noted that this method is heavily influenced by various external conditions such as pressure or the angle of the transducer^61^. Taken together the aforementioned advantages over other methods, the US measurement of TLFD presented here, are a promising tool for clinical purposes, firstly for identifying patients with acute LBP and secondly for monitoring treatment progress in the context of an intervention. In addition, it could be helpful to identify TLFD restrictions as a possible risk factor for the development of chronic LBP, as only one third of these patients recover within three months^5^. Swelling and thickening of the TLF, e.g. due to micro-injuries or hypoxia, could lead to densification, inflammation, and a more adhesive behavior of the gliding surfaces between the fascial layers^25,62,63^. This is probably an additional parameter in the complex multifactorial cascade of LBP development and most likely already present in the early stages^17^. Brandl et al.^16,64^ demonstrated that artificially induced aLBP is associated with swelling and typical pain patterns that can be localized in the fascial tissue. Monitoring this process is therefore a promising method in the treatment of LBP. This should be seen in the absence of alternative imaging techniques such as computed tomography or magnetic resonance imaging, whose suitability for the dynamic assessment of pathologically altered anisotropic tissue material may be limited^54^. Here, even the relatively high MDC, which was determined on the basis of inter-rater reliability, exceeds the accuracy of most other conventional diagnostic procedures^55–57^.

### Study limitations

This study has some limitations. Firstly, the study groups were not matched, which would have led to a higher internal validity. However, the comparison of the baseline characteristics showed no significant differences between the groups. The use of an independent second group could therefore have had the advantage of higher external validity in real clinical settings^65^. Secondly, the exact degree of trunk flexion was not measured objectively. It can therefore not be said that participants achieved the same movement amplitudes. Furthermore, it is possible that aLBP patients used different movement and muscle recruitment strategies to reach the starting and ending positions. An analysis of surface electromyographic data of the erector spinae muscle at L1 from a previous study showed muscle activity during US measurement in 2 of 20 participants that did not influence group differences in TLFD^29^. Third, the raters had varying levels of experience with US examination, particularly testing the TLF. We only considered the intra-rater reliability of the experienced rater as we assumed that this would be the case in most clinical situations. However, future work could include the assessment of the intra-rater reliability of an inexperienced rater for extended comparability. The raters were further only familiarized with the method for half an hour at the beginning of the study. Therefore, this reliability study may be of limited use for experimental purposes. The design aimed to mimic and provide data for an easy-to-perform examination in daily clinical practice. There is a high probability that the ICC's and MDC's determined in this trial would be significantly improved by taking the aforementioned points into account, but this was not the focus of this study and is a task for future work.

The US method for assessing TLFD in this study has excellent intra-rater and good inter-rater reliability. It was able to distinguish aLBP patients from healthy individuals and showed higher discriminative validity than one-dimensional assessment methods. The intra-rater MDC was below the cut-off point for discrimination. Therefore, this method could be useful in monitoring treatment progress after an intervention. It is promising to explore whether TLFD restrictions could be a potential risk factor for the development of chronic LBP and thus complement diagnostics in the complex multifactorial cascade etiology of this disease.

## Data Availability

The datasets that will be used and/or analyzed during the current study will be available from the corresponding author on reasonable request.
